# Vitamin D as an Adjunctive Treatment to Standard Drugs in Pulmonary Tuberculosis Patients: An Evidence-Based Case Report

**DOI:** 10.1155/2019/5181847

**Published:** 2019-06-20

**Authors:** Diajeng Ayesha Soeharto, Diana Ashilah Rifai, Stella Marsudidjadja, Aisha Emilirosy Roekman, Chadijah Karima Assegaf, Melva Louisa

**Affiliations:** ^1^Medical Student, Faculty of Medicine, Universitas Indonesia, Indonesia; ^2^Department of Pharmacology and Therapeutics, Faculty of Medicine, Universitas Indonesia, Indonesia

## Abstract

**Background:**

Vitamin D has a prominent role in the body's innate immunity as it is important in the maintenance of macrophages and monocytes and its function in defending against infections.* In-vitro* studies have established vitamin D's potential role in tuberculosis (TB) infection, in that it restricts* Mycobacterium tuberculosis *growth, thus implying the potential benefit of vitamin D as an adjunctive treatment for TB. However, clinical trials and reviews have contradicting findings regarding the true clinical efficacy of adjunctive vitamin D, particularly in reducing the sputum conversion rate (SCR).

**Objective:**

This study aims to update the current evidence regarding vitamin D supplementation as an adjunctive treatment in achieving the smear sputum conversion rate (SCR) among pulmonary TB patients.

**Method:**

A comprehensive search was conducted in October 2018 in PubMed-NCBI, MEDLINE-OVID, SCOPUS-Elsevier, and Cochrane. The selection of studies was done as per the predetermined inclusion and exclusion criteria of this EBCR and resulted in the inclusion of 11 eligible studies (8 RCTs and 3 systematic reviews). The selected studies were then critically appraised for their validity, importance, and applicability according to the CEBM (Centre for Evidence-Based Medicine) appraisal tools.

**Results:**

Overall, most of the trials showed no statistically significant changes in terms of the proportion of TB patients with a negative sputum smear conversion in the group treated with an adjunctive therapy vs. the group treated with standard antituberculosis therapy alone. Only one trial showed significant results, which was conducted in a population of TB patients with vitamin D deficiency. Furthermore, overall the reviews showed no significant change in the 8-week sputum smear conversion after treatment within the group given vitamin D in comparison to those who were not.

**Conclusion:**

Vitamin D as adjunctive therapy in TB patients shows no clinical improvement in terms of sputum conversion to antituberculosis management.

## 1. Introduction

A woman came to the clinic with her 41-year old husband. Her husband was diagnosed with pulmonary tuberculosis (TB) last month and had come back for a follow-up consult. The husband reported that he had been routinely taking the prescribed anti-TB drugs since then. During the consultation, she mentioned that she read some information online regarding the benefits of vitamin D supplements for TB patients. She was wondering whether or not vitamin D supplements could improve her husband's treatment outcome if he were to take them along with his anti-TB drugs.

## 2. Background

Tuberculosis infection (TB) is one of the top ten causes of death worldwide and is due to* Mycobacterium tuberculosis *(Mtb) infection. Although this bacterium can affect numerous organs, the respiratory system remains the most prominently affected. TB is a contagious disease, and air serves as the main mode of transmission [1].

Due to its ease of transmission, early prevention and treatment are crucial. Early diagnosis can be done since a susceptible patient is suspected of having a TB infection. The examination includes taking a history, physical examination, radiological chest examination, and the laboratory culture of* Mycobacterium tuberculosis *by culture of sputum or tissue biopsy. A sputum smear test can also be conducted to assess the degree of infection. Treatment for tuberculosis includes a combination of drugs administered for at least 6 months, and the conversion of sputum smear test (SSC) from positive to negative indicates successful treatment and generally occurs after two months of treatment [2, 3]. The two-month sputum conversion rate is currently considered as the preferred endpoint because this marker has a significant correlation with combined response rates, relapse, and intermediate bacteriological endpoint [2]. Standard treatment for TB consists of 4 antituberculosis drugs, which are isoniazid, rifampin, pyrazinamide, and either ethambutol or streptomycin. This regimen is given for 2 months with 60 doses, followed by 4 months of treatment with only isoniazid and rifampin with 120 doses. The choice of treatment depends on the patients' drug susceptibility and the response following treatment [1, 4].

Vitamin D has a vital role in the body's natural defense mechanism towards infection since it promotes the role of macrophages and monocytes which is important in pathogenesis [5]. In infection caused by* Mycobacterium tuberculosis *(Mtb), calcidiol, the major circulating metabolite of vitamin D, supports the induction of innate antimicrobial immune response by causing Mtb growth restriction. Calcidiol is metabolized by CYP27B1 into its active form, calcitriol [6]. Calcitriol exerts its antimicrobial activity by modulating the reactive oxygen intermediates, the induction of antimicrobial peptides, and autophagy [7]. Calcitriol plays a key role in the maintenance of immune homeostasis and in tuberculosis and works indirectly by suppressing MHC class II and directly suppresses IFN-*γ* and IL-2 from CD4 Th1 cells [8]. Coussens et al. have also shown that high dose of vitamin D supplementation suppresses antigen-stimulated proinflammatory cytokine responses and accelerates the resolution of IL-4, CC chemokine ligand 5, and IFN-*α* secretion during the administration of antituberculosis drugs [9].

Thus, some studies have suggested adjunct vitamin D supplementation to enhance the response to antituberculosis therapy [10]. However, several studies have differing results regarding the impact of vitamin D as adjunctive therapy to TB patients [11]. Therefore, the purpose of this article is to update and clarify the impact of adjunct vitamin D supplementation in achieving an early smear sputum conversion rate among pulmonary TB patients.

## 3. Method

### 3.1. Search Strategy

A comprehensive literature search was conducted in October 2018 on four electronic databases: PubMed-NCBI, MEDLINE-OVID, SCOPUS-Elsevier, and Cochrane by using keywords and Mesh terms as follows: “Tuberculosis,” “Vitamin D,” (Sputum OR “Sputum Conversion Rate” OR SCR). The keywords were then incorporated with Boolean operators into (“Tuberculosis”[MeSH]) AND “Vitamin D”[MeSH] AND (Sputum OR “Sputum Conversion Rate” OR SCR), or an equivalent of that to suit each database.

**Figure 1:**
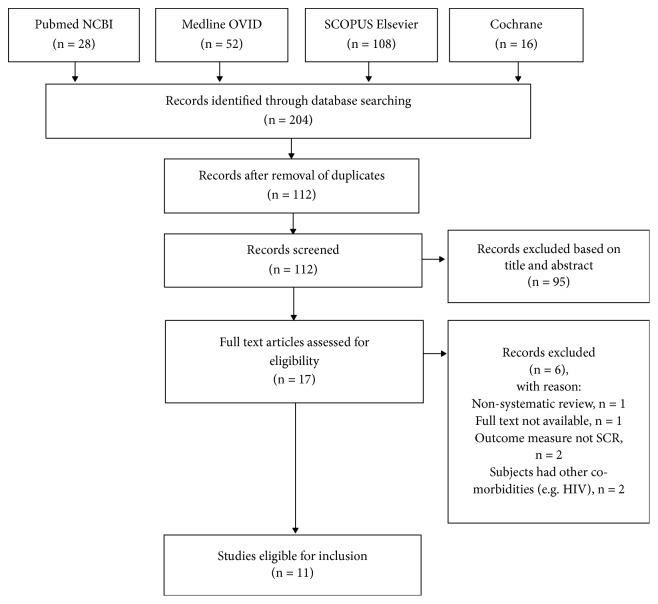
PRISMA flowchart outlining the process of article selection.

A search limitation was not applied in this search strategy to avoid overlooking any articles. The search results', from all four electronic databases, all duplicated articles were first removed, and the results were then screened by evaluating the title and abstract with regard to the eligibility criteria of this EBCR (outlined in Figure 1). The final inclusion of studies was determined by examining each full article and evaluating whether or not the study satisfied the inclusion criteria.

### 3.2. Eligibility Criteria

The targeted population was patients with a Tuberculosis infection who were undergoing anti-TB therapy with the sputum smear conversion rate as the outcome measure. The inclusion criteria for the research evidence were randomized controlled trials (RCT), systematic reviews, and meta-analysis studies which compare two different interventions: standard combination anti-TB drug therapy versus standard combination anti-TB drug therapy with vitamin D supplementation. The participants of the study were expected to have the absence of any predisposing illness (such as Type II Diabetes Mellitus). Moreover, the study with an adjuvant therapy to combine with vitamin D supplementation for the participants in the intervention group was considerably biased and thus excluded. Articles were excluded if the papers included patients with comorbidities or secondary chronic infections such as HIV, HBV, or HCV, they were animal or* in-vitro* studies, the full-text was not available, and if the study did not incorporate a sputum smear conversion as the end-point measure.

### 3.3. Critical Appraisal

The CEBM (Centre for Evidence-Based Medicine) appraisal tools [22] were used to systematically evaluate the quality of evidence included in this EBCR. CEBM appraisal sheets for RCTs and systematic review sheets were used with respect to each study type. As for the RCT critical appraisal sheet, the quality was evaluated within the domains of internal validity (mainly focusing on randomization, blinding, and comparability) and clinical importance (study findings). Furthermore, systematic reviews were evaluated with regard to the domains of a comprehensive and appropriate search strategy, validity, and heterogeneity of included studies, as well as the conclusion drawn by the review.

## 4. Results

### 4.1. Search Findings

As shown in Figure 1, the initial search of four electronic databases using the aforementioned search terms yielded 204 potentially relevant studies (Table 1) from which 90 duplicate studies were removed. The remaining 112 studies were screened based on the title and abstract, where 97 were excluded as they either did not study adjunctive vitamin D therapy in TB patients, included patients with other comorbidities, did not evaluate SCR, or were not RCTs, systematic reviews, or meta-analyses. The remaining 17 potentially relevant studies were assessed for eligibility by examining the full-text articles, with regard to the EBCR eligibility criteria. From this, a total of 6 studies were excluded: one study was not a systematic review, one did not have the full-text available, two did not include the SCR as its outcome measure, and two included subjects with other comorbidities. Thus, a total of 11 studies, consisting of three systematic reviews and eight RCTs, were selected and considered eligible for inclusion in this EBCR.

### 4.2. Critical Appraisal

Out of the eight RCT studies, 6 completely fulfilled the internal validity criteria in the CEBM RCT appraisal tool by the University of Oxford [22] (Table 2). Primarily, the studies (7 out of 8) found that there was no significant difference between adjunctive vitamin D3 and the control treatment in achieving a negative sputum culture result by the end of the fixed time (2 months), as portrayed by the 95% CI of the RCTs which crossed the line of no effect, except for the study by Afzal et al. [12].

The number needed to treat (NNT) varied considerably (as shown in Table 2), with 10 and 660 as the minimum and maximum value, respectively, and a median of 43 patients as needed to treat in order for one good outcome of sputum conversion. Notably, the NNT varies greatly from study to study, and although this may be suggestive of the varied quality and design of one study to the other, this range of NNT is also typical of trials involving vitamins and preventive therapy in general; thus the values in our appraised studies are as expected.

Overall, the three included reviews were of varying quality (Table 3). While all three reviews used appropriate methods in searching for and including evidence, Wallis et al. [20] failed to assess the quality of the included studies and thus were unable to fulfill the criteria for the included study validity. Furthermore, since Wallis et al.'s study was merely a systematic review and did not include a pooled quantitative analysis of the studies, the review failed to fulfill the criteria for the resulting similarity. Wang et al. [21] and Wu et al. [10] were both meta-analyses of relatively good quality. The two differed in that Wang et al. showed that the included studies had no signs of heterogeneity, while Wu et al. showed considerably high levels of heterogeneity; however, it is worth noting that in the Wang et al. pooled quantitative analysis regarding vitamin D and SCR conversion rates, they did not analyze all five of the included studies in the review and also failed to provide a justification as to why some studies were excluded in the quantitative analysis [21].

### 4.3. Vitamin D Mode of Administration and Dose

Across all the trials, vitamin D was given concurrently with typical ATT, and the comparison groups were given ATT alone or along with the control. Vitamin D administered orally in 7 studies, where Ganmaa et al. [14] gave four 3.5 mg tablets with a total dose of 140,000 IU of vitamin D3, Nursyam et al. [17] gave oral tablets of 0.25 mg per day for six weeks, Ralph et al. [18] gave one tablet every four weeks of “Calciferol Strong®” (50,000 IU, 1250 mcg), and Tukvadze et al. [19] gave 1.25 mg (50,000 IU) of vitamin D3. Other oral modes of administration included the use of 2.5 mg of vitamin D oil (10,000 IU) given every fortnight for 6 weeks in the study by Daley et al. [13], Martineau et al. [15] used oral vigantol oil, which is similar to that of Mily et al. [16] who used the same type of oil, but at 5,000 IU given once daily. Vitamin D was given intramuscularly in one study by Afzal et al. [12], in which four separate large doses of 10,000 IU were given every fortnight (Table 4). As for the included reviews, the study populations were treated similarly in terms of vitamin D administration to that of the aforementioned trials. All three reviews included both oral and intramuscular administration of vitamin D and had a range of doses that differed in each study; they were typically given during the intensive phase of TB treatment (8 weeks) and administered every one to two weeks (see Table 5).

### 4.4. Vitamin D and Sputum Conversion Rate

The included RCTs, as shown in Table 4, show that the outcome measure of all the studies was similar and monitored the sputum conversion from positive to negative at different times. For instance, Ralph et al. [18] measured this conversion at the 4th week and followed up at the 8th week after intervention, whereas Tukvadze et al. [19] and Ganmaa et al. [14] identified the changes at the 8th week. In addition, three other studies by Nursyam [17], Mily et al. [16], and Afzal et al. [12] did repeated examinations of the sputum conversion. Although the treatment follow-ups were done at different frequencies and timepoints, all the studies have SCR endpoints at 8 weeks. Thus, the calculation of NNT and the 95% CI are based on SCR at the 8th week after treatment.

Almost all the results of these RCTs showed no significant impact of delivering vitamin D as adjunctive therapy along with ATT to the early sputum conversion rate compared with the standalone consumption of ATT. Conversely, one study by Afzal et al. [12] did show a small benefit of vitamin D consumption compared to the control group to those receiving vitamin D (11.7% vs. 1.7%, p = 0.028). Albeit, this study concerned only vitamin D deficient patients. Moreover, Nursyam et al. [17] also showed significant differences in the proportion of negative SCR observed between vitamin D and the placebo group (100% vs. 76.7%, p = 0.002) only at the 6th week after treatment; however at the 8th week, only 1 patient in the placebo group did not have an SCR (100% vs. 96.9%; p = 0.99). The reviews, on the other hand, had contradicting results (see Table 5). While the review by Wang et al. was suggestive of a significantly higher proportion of sputum conversion in those treated with vitamin D (OR = 1.21, 95% CI = 1.05–1.39, p = 0.007), this finding is not in line with the results of the other two newer reviews. The qualitative analysis by Wallis et al. analysis concluded that vitamin D supplements were not of benefit in terms of the earlier sputum conversion, and this is further corroborated with the findings of Wu et al.'s pooled qualitative analysis that showed that vitamin D supplementation had no influence on the improvement of sputum smear-negative conversion rates (RR = 0.99; 95% CI = 0.91 to 1.07;* p *= 0.77). Additionally, Wallis et al. also mentioned potentially concerning incidences of the side-effects along with vitamin D supplements, although they were regarded as safe overall.

## 5. Discussion

Across all of the appraised RCTs, only the study by Afzal et al. [12] had significant clinical importance as indicated by the confidence interval and relative risk (as shown in Table 2). We thought that the main reason that Afzal et al. showed significant effects was that the study population is vitamin D deficient, while other studies administered vitamin D despite their vitamin D status. We thought that the mode of administration of vitamin D might play a role in the difference in results, particularly since Afzal et al. used an intramuscular injection to administer vitamin D. The pharmacokinetic characteristics can potentially highly influence sputum conversion results and thus would differ in patients given an intramuscular injection compared to those given oral supplementation. Gupta et al. studied the pharmacokinetic difference of oral versus intramuscular vitamin D in healthy adults with vitamin D deficiency and showed that both modes of administration were effective in correcting hypovitaminosis D. However, Gupta et al. showed that intramuscular overtime (after 6th to 12 weeks of treatment) serum 25-hydroxyvitamin D was better maintained by intramuscular injection rather than oral administration [23]. Moreover, since the population in the study by Afzal et al. focused only on correcting vitamin D deficiency rather than vitamin D supplements in the general population, inferences of the study results into the general population cannot be done. Further investigation into the use of vitamin D intramuscular therapy in the form of RCTs and meta-analyses needs to be conducted in order to establish its true clinical benefit. With the current state of evidence, it would be inefficient to add adjuvant therapy if drastic benefits cannot be reaped from it. Use of IM vitamin D adjuvant therapy is not applicable in some countries; taking into consideration its questionable benefit, the added cost, invasiveness of injections, and especially the added burden of treatment for TB patients (considering that TB is a disease that deals majorly with issues of noncompliance due to the need for multiple drugs over an extended period of time), it cannot be recommended.

Patient selection criteria, dose, mode of vitamin D administration, and methodological differences between the studies might also contribute to the differences in results, which might attenuate the true clinical effect of vitamin D. Most of the studies were done using a small number of subjects, so they were considered as underpowered. The largest study was done by Ganmaa et al. in 390 Mongolian people. The study by Ganmaa et al. confirmed that the genetic variation in vitamin D metabolizing enzymes CYP27B1 modified the protective effect of vitamin D [14].

Different sputum sampling methods of SCR sampling might also contribute to the difference in the results. The smear samples used were variables between trials. Sputum microscopy is the most common method used to determine SCR [14, 18, 19], while Daley et al. [13] and Martineau et al. [15] used fluorescent microscopy. The fluorescent microscopy method is higher in sensitivity and faster than conventional microscopy, while sputum microscopy is time-consuming and has a lower sensitivity in HIV coinfection [3].

In interpreting the systematic review by Wallis et al. [20], it is important to take into consideration the differences in the eligibility criteria of the included studies. Although the stated inclusion criteria were comparable to that of this EBCR (where subjects with comorbidities were to be excluded), the review by Wallis et al. had included one study that we had excluded during our selection process due to the inclusion of patients with HIV infection. The immunosuppressive condition of participants might act as a significant confounding factor to the sputum conversion differences in adjunctive vitamin D therapy. Furthermore, another point worth noting is the use of an odds ratio instead of relative risk in the review by Wallis et al. and Wu et al. which was not justified by the respective authors. Considering that the population and the incidence of the outcomes of the studies were not controlled, the use of an odds ratio would tend to show an overestimation of the true odds and thus would not provide an accurate estimate of the odds.

## 6. Conclusion

Adjunctive vitamin D therapy shows no clinical improvement (sputum conversion) in antituberculosis management. It is not necessary to add oral vitamin D to the current antituberculosis regimen that the patient takes. Further studies regarding vitamin D adjunctive therapy in nondeficient TB patients are needed in order to establish the true effect of vitamin D in the general population of TB patients, particularly evaluating its safety and efficacy.

## Figures and Tables

**Table 1:** Search strategy applied in searching for evidence in the four electronic databases.

Database	Search Strategy	Hits	Selected Articles
PubMed NCBI	(“Tuberculosis”[Mesh]) AND “Vitamin D”[Mesh] AND (Sputum OR “Sputum Conversion Rate” OR SCR)	28	7
Medline OVID	1. TUBERCULOSIS/or Tuberculosis.mp. 2. Vitamin D/or Vitamin D.mp. 3. (“Sputum Conversion Rate” or Sputum*∗* or SCR).mp. [mp=title, abstract, original title, name of substance word, subject heading word, floating sub-heading word, keyword heading word, protocol supplementary concept word, rare disease supplementary concept word, unique identifier, synonyms] 4. 1 AND 2 AND 3	52	3
SCOPUS Elsevier	TITLE-ABS-KEY (Tuberculosis AND “Vitamin D” AND (Sputum OR “Sputum Conversion Rate” OR SCR))	108	1
Cochrane	(MeSH descriptor: [Tuberculosis]: explode all trees) AND (MeSH descriptor: [Vitamin D]: explode all trees) AND Sputum	16	0

**Table 2:** Critical appraisal of RCTs using the CEBM tool by the University of Oxford.

*Author*	No. of Participants	Level of Evidence	*Internal Validity∗*					*Importance∗∗*				
			Randomisation	Baseline Similarity	Equally Treated	Intention to Treat	Blinding	*RR*	*ARR (*%)	*RRR (*%)	*NNT*	*95*%* CI*
Afzal et al. (2018) [12]	120	2	+	+	+	+	-	1.11	10.0	11.3	10	(1.05, 1.26)
Daley et al. (2015) [13]	247	2	+	+	+	+	+	1.02	12.0	17.4	83	(0.86, 1.18)
Ganmaa et al. (2017) [14]	390	2	+	+	+	+	+	1.07	49.4	6.7	20	(0.96, 1.18)
Martineau et al. (2011) [15]	108	2	+	+	+	+	+	0.98	15.1	18.8	66	(0.79, 1.17)
Mily et al. (2015) [16]	127	2	+	+	+	+	+	1.15	94.7	15.4	11	(0.9, 1.4)
Nursyam EW (2006) [17]	67	2	+	+	+	+	+	1.03	3.0	3.1	34	(0.9, 1.1)
Ralph et al. (2013) [18]	79	2	+	-	+	+	+	0.99	0.4	0.7	222	(0.64, 1.34)
Tukvadze et al. (2015) [19]	199	2	+	+	+	+	+	1.00	0.2	0.2	660	(0.88, 1.12)

*∗* Internal validity indicated by + stated clearly in the article; - not being done; ? not stated clearly

*∗∗* Importance was calculated based on the SCR at the 8th week of treatment.

**Table 3:** Critical appraisal of the systematic reviews using the CEBM tool by the University of Oxford.

Author	Clear PICO	FAAT				Results		
		Appropriate searching	Appropriate inclusion criteria	Study validity	Result similarity	Total 95% CI	Heterogeneity	Overall effect
Wallis et al. (2016) [20]	+	+	+	−	−	−	−	−
Wang et al. (2018) [21]	+	+	+	+	+	RR 0.9 [0.91, 1.07]	Chi^2^ = 1.27, df = 2 (p = 0.53), I^2^ = 0%	Z = 0.30 (p = 0.77)
Wu et al. (2018) [10]	+	+	+	+	−	OR 1.21 [1.05, 1.39] OR 1.22 [1.04, 1.43]	Chi^2^ = 13.25, df = 3 (*p* = 0004), I^2^ = 77%; Chi^2^ = 11.15, df = 3 (*p* = 0.01), I^2^ = 73%	Z = 2.69 (p = 0.007); Z = 2.41 (p = 0.77)

FAAT indicated by + stated clearly in the article; − not being done; ? not stated clearly.

**Table 4:** Summary of included randomized controlled trials.

Author	Study population	Vitamin D (method of administration, dose)	Outcome measure	Results
Afzal et al. (2018)	Adult newly diagnosed TB patients with vitamin D deficiency in Lahore, Pakistan (n = 120)	Intramuscular injection of vitamin D (four doses of 100,000 IU) after 14 days during the intensive-phase	Sputum examination repeated at 2nd, 4th, 6th, 8th, 10th, and 12th weeks. Early SCR shown by the conversion of AFB to negative four weeks after treatment initiation	The intervention group who received vitamin D was 1.11 times as likely to have earlier SCR during the 12-week examination; significant results of positive SCR seen in control vs vitamin D (11.7% vs 1.7%, p = 0.028)

Daley et al. (2015)^+∗^	Adult TB patients in Tamil Nadu, India (n = 247)	Four doses of adjunctive 2.5 mg oral vitamin D3 oil (100,000 IU) at weeks 0, 2, 4, and 6	Sputum culture conversion (time to first negative smear), estimate in median time to culture conversion	No significant difference of the median time to culture conversion between vitamin D and the placebo group (RR = 1.02)

Ganmaa et al. (2017)^∗^	Adult TB patients in Ulaanbaatar, Mongolia (n = 390)	Four oral doses of 3.5 mg (140,000 IU) vitamin D3 (each dose is 4 tablets containing 875 *μ*g)	The proportion of participants with negative sputum culture at week 8	The administration of vitamin D versus the placebo did not influence the proportion of participants with sputum culture conversion at week 8 (Adjusted OR = 1.47, 95% CI = 0.88–2.45, p = 0.14)

Martineau et al. (2011)^+-*∗*^	Adult TB patients in London, UK (n = 126)	Adjunctive oral vigantol oil (2.5 mg)	Proportion of TB patients with a negative sputum culture at 56 days as the secondary outcome	Vit D3 group is 0.98 times as likely to achieve the negative sputum culture result at 56 days than the placebo group (p = 0.85)

Mily et al. (2015)^∗^	Adult TB patients in Dhaka, Bangladesh (n = 288)sd	Adjunctive oral vigantol oil (5000 IU vitamin D3) once daily	Proportion of TB patients became culture negative and major clinical endpoints at weeks 4 and 8 as primary outcomes	Odds of sputum culture being negative at week 4 are 2.20 times higher in vit D3 group (95% CI = 1.07–4.51, p = 0.032), and 7.26 higher at week 8 (95% CI = 0.90–25.50, p = 0.062)

Nursyam EW (2006)^+*∗*^	Adult TB patients visiting the Pulmonary Clinic of RSCM from Jan 1st to Aug 31st in 2001 (n = 67)	Oral Vitamin D tablets (0.25 mg/day) given in initial 6 weeks of Anti-TB drugs therapy (2RHZE/4H)	Negative AFB at the beginning of trial, on the 6th and 8th week If AFB (+) test, another AFB test on 12th week	Significant differences in the proportion of negative SCR observed between vitamin D and the placebo group (100% vs 76.7%, p = 0.002) at 6th week; however, at the 8th week the differences of SCR versus the placebo were 100% vs 96.9% (p = 0.99)

Ralph et al. (2013)^−^	Adult newly diagnosed PTB patients in Timika, Southern Papua, Indonesia (n = 79)	Oral adjunctive active cholecalciferol (vitamin D3, “Calciferol Strong®”) 50,000 IU (1250 mcg, 1 tablet) 4 times a week	Negative sputum culture on liquid medium at week 4 and a composite clinical severity score at week 8 (including presence/absence of sputum and sputum smear conversion time)	Risk difference of vitamin D versus vitamin D-Placebo: 7%, 95% CI [−9, 22]. Vit D did not, at dose administered and with the power attained, affect the outcomes of TB

Tukvadze et al. (2015)^+-*∗*^	Adult TB patients in Tbilisi, Georgia (n = 199)	Adjunctive oral 1.25 mg vitamin D3 (50,000 IU) 3 times weekly for 8 consecutive weeks, and 50,000 IU oral vitamin D3 every 2 weeks for an additional 8 weeks	Sputum culture conversion at 8th week as the secondary clinical outcome	No significant difference between the high dose vitamin D3 and the placebo groups in achieving a negative sputum culture (HR = 1.20, 95% CI = 0.80−1.88, p = 0.004)

^+^included in Wallis et al., ^−^  included in Wang et al., ^∗^ included in Wu et al.;

PTB: Pulmonary Tuberculosis; TB: Tuberculosis; SCR: Sputum Conversion Rate.

**Table 5:** Summary of the included systematic reviews and meta-analyses.

Author	Study population	Vitamin D (Method of administration, dose)	Results	Comments
Wallis et al. (2016)	Adult patients with PTB	Vitamin D given orally or intramuscularly at total doses of 2.5 mg to 30 mg.	In general, vitamin D was not shown to reduce the treatment time (except for one study). Supplementation was found to be well-tolerated and safe. Concern for the paradoxical reaction in the vitamin D subjects (i.e., disease worsening despite microbiological improvement) resulting in surgical or radiological intervention or deaths.	The authors argued that the paradoxical effects of vitamin D are due to the different baseline characteristics, mainly the extent of vitamin D deficiency. The authors highlighted the possibility of genetic polymorphisms in vitamin D receptor or enzymes involved in vitamin D metabolism
Wang et al. (2018)	Adult patients with PTB	Vitamin D given intramuscularly (50,000 IU - 60,000 IU) or orally 2.5 mg every 1–2 weeks)	Vitamin D supplementation showed no influence on the improvement of sputum smear-negative conversion rates (RR = 0.99; 95% CI = 0.91 to 1.07; *p *= 0.77). There was no significant difference in the serious adverse events between the vitamin D supplementation group and the placebo group (RR = 1.03; 95% CI = 0.25 to 4.31; *p *= 0.96).	The authors stated that a higher dose and longer treatment period of vitamin D are needed in order to achieve a favorable treatment effect.
Wu et al. (2018)	Patients above 16 years old that were newly diagnosed with PTB and who were on initial anti-TB treatment	Vitamin D given at different doses ranging from 1000 IU/day to 600,000 IU/month at different intervals	Vitamin D supplementation increased the sputum smear proportion conversion (OR = 1.21, 95% CI = 1.05–1.39, *p *= 0.007) and the sputum culture conversion (OR = 1.22, 95% CI = 1.04–1.43, *p *= 0.02).	Although this study found that vitamin D is beneficial in increasing the overall effect of sputum smear conversion, no difference was found for the conversion at the 8th week. The authors also reported that vitamin D does not improve other parameters such as TB score, CRP, ESR, and blood indices.

PTB: Pulmonary Tuberculosis; CRP: C-reactive protein; ESR: erythrocyte sedimentation rate.
